# Epidemiology of Rounding Error

**DOI:** 10.3390/medicina60122105

**Published:** 2024-12-22

**Authors:** Jimmy T. Efird

**Affiliations:** 1 Cooperative Studies Program Coordinating Center, VA Boston, Lafayette City Center, 2 Avenue de Lafayette, Boston, MA 02111, USA; jimmy.efird@va.gov or jte38@case.edu; 2 Department of Radiation Oncology, School of Medicine, Case Western Reserve University, Cleveland, OH 44206, USA

**Keywords:** accuracy, data truncation, epidemiology, precision, relative effect estimation, risk reduction, rounding error

## Abstract

This work represents a significant contribution to understanding the importance of appropriately rounding numbers with minimal error. That is, to reduce inexact rounding and data truncation error and simultaneously eliminate unintentional misleading findings in epidemiological studies. The rounding of numbers represents a compromise solution that attempts to find a balance between the loss of information from reporting too few significant digits versus retaining more digits than necessary. Substituting a rounded number for its original value may be acceptable and practical in many applied situations if an adequate degree of accuracy is retained. On the other hand, numeric error may result from improper rounding or data truncation which, in effect, compromises the credibility of study findings and may lead to a false sense of discovery. Performing complex computations on such values, especially when sequential or composite operations are involved, can lead to error propagation and inaccurate results. Having an overall awareness of the nature and impact of rounding error, including preventive actions, can contribute greatly to the integrity of research, yielding more reliable and accurate conclusions. Heuristic examples are provided to illustrate the consequences of rounding and data truncation error in epidemiology studies, specifically those pertaining to relative effect estimation.

## 1. Introduction

Often, epidemiologic data may be reported with more decimal precision than is sensible or practical, and the excess may even be distracting to readers [1]. Rounding provides a real-world way to simplify the presentation of study findings and reduce spurious accuracy. This is especially true when the extra digits are unnecessary or inappropriate given the inexactness of the underlying measurement tool [2]. The process involves exchanging a more exact value with one of lower accuracy while minimizing the practical loss of validity. Yet, the imprecision introduced by rounding or data truncation may result in erroneous findings and the misinterpretation of results—especially when performing complex epidemiologic analyses that entail the accumulation of rounding error. Researchers need to be vigilant to not report values that contain too few significant digits. Equally important, they should avoid providing more significant figures than are warranted.

Guidance on this topic is not well-established in the literature, even though inexact rounding and data truncation error may inadvertently mislead study findings. This is particularly true when comparing relative effects between study groups, estimating risk reduction, or conducting a meta-analysis involving multiple risk effects.

The aim of this research is the presentation of preliminary concepts and techniques for rounding and data truncation. Didactic examples are provided with the intention of better understanding the importance and implications of this topic in the field of epidemiology.

## 2. Preliminaries

### 2.1. Confidence Interval for the Ratio of Two Relative Effect Estimates

Relative effect estimates (REEs) are a type of epidemiologic risk measure that compares the exposure event attributes between an index and referent group. This class of estimates, which includes odds ratios (ORs), risk ratios (RRs), and hazard ratios (HRs), are commonly used to evaluate the relative importance of a predefined condition (risk) versus the absence of this factor, with respect to the occurrence of a particular disease or result.

Let ${\hat{\vartheta }}_{i}\left(i=1,\;2\right)\;$ denote the REEs corresponding to two independent samples, having a common referent (control) arm. The large sample $\left(1-\alpha \right)100\%\;confidence\;interval$ (CI) for the ratio $\left(\frac{{\hat{\vartheta }}_{1}}{{\hat{\vartheta }}_{2}}\right)$ may be estimated as

(1) $${CI}_{\alpha }\left(\frac{{\hat{\vartheta }}_{1}}{{\hat{\vartheta }}_{2}}\right)=e^{log\left(\frac{{\hat{\vartheta }}_{1}}{{\hat{\vartheta }}_{2}}\right)\pm z_{\left(1-\frac{\alpha }{2}\right)}SE\left[log\left(\frac{{\hat{\vartheta }}_{1}}{{\hat{\vartheta }}_{2}}\right)\right]}$$

(2) $$=e^{log\left(\frac{{\hat{\vartheta }}_{1}}{{\hat{\vartheta }}_{2}}\right)\pm z_{\left(1-\frac{\alpha }{2}\right)}\sqrt{{\left(SE\left[log\left({\hat{\vartheta }}_{1}\right)\right]\right)}^{2}+{\left(SE\left[log\left({\hat{\vartheta }}_{2}\right)\right]\right)}^{2}}},$$

where $\left(SE\right)$ represents the respective standard error estimate of the term within the parentheses and $\left(\alpha \right)$ is defined as the probability of rejecting the null hypothesis when it is true [3]. Here, $log\left(\frac{{\hat{\vartheta }}_{1}}{{\hat{\vartheta }}_{2}}\right)$ is referred to as the log difference, since the logarithm of a ratio is equivalent to the logarithm of the numerator minus the logarithm of the denominator.

The z-statistic and *p*-value for testing that two REEs differ at the α-level of statistical significance are given as

(3) $$z=\frac{log\left(\frac{{\hat{\vartheta }}_{1}}{{\hat{\vartheta }}_{2}}\right)}{\sqrt{{\left(SE\left[log\left({\hat{\vartheta }}_{1}\right)\right]\right)}^{2}+{\left(SE\left[log\left({\hat{\vartheta }}_{2}\right)\right]\right)}^{2}}},$$

and

(4) $$P=2\cdot \left\{1-\Phi \left(z\right)\right\},$$

where

(5) Φz=∫−∞ze−x222·πdx.

If ${CI}_{\alpha }\left(\frac{{\hat{\vartheta }}_{1}}{{\hat{\vartheta }}_{2}}\right)$ excludes unity, a result is declared to be statistically significant, equivalent to an α-level *p*-value.

### 2.2. Risk Reduction on the Relative Effect (Log-Difference) Scale

When $\left({\hat{\vartheta }}_{1}>{\hat{\vartheta }}_{2}\right)$, risk reduction on a log difference scale is computed as $\left(\frac{{\hat{\vartheta }}_{1}}{{\hat{\vartheta }}_{2}}-1\right),$ and vice versa when $\left({\hat{\vartheta }}_{2}>{\hat{\vartheta }}_{1}\right),\;$ i.e., $\left(\frac{{\hat{\vartheta }}_{2}}{{\hat{\vartheta }}_{1}}-1\right).$ If $\left({\hat{\vartheta }}_{1}={\hat{\vartheta }}_{2}\right)$, a null result is obtained for the risk reduction. In the case of RRs, the risk reduction between two samples is determined directly. Under appropriate conditions, ORs and HRs may be used to approximate risk within each sample.

### 2.3. Standard Error (SE)

Three methods exist for computing the standard error of $\left[log\left({\hat{\vartheta }}_{i}\right)\right]$. The default approach (Method 1) is to divide the width of the 95% CI for $log\left({\hat{\vartheta }}_{i}\right)$ by twice the $\left(1-\frac{\alpha }{2}\right)100\%$ percentile of a standard normal distribution. The other two methods entail symmetrically pivoting off the midpoint of the above width with respect to the lower and upper confidence bound.

-Method 1

Re-expressing $\;SE\left[log\left({\hat{\vartheta }}_{i}\right)\right]$, one observes that

(6) $$SE\left[log\left({\hat{\vartheta }}_{i}\right)\right]=\frac{2SE\left[log\left({\hat{\vartheta }}_{i}\right)\right]z_{\left(1-\frac{\alpha }{2}\right)}}{2z_{\left(1-\frac{\alpha }{2}\right)}}$$

(7) $$=\frac{log\left({\hat{\vartheta }}_{i}\right)+z_{\left(1-\frac{\alpha }{2}\right)}SE\left[log\left({\hat{\vartheta }}_{i}\right)\right]-\left\{log\left({\hat{\vartheta }}_{i}\right)-z_{\left(1-\frac{\alpha }{2}\right)}SE\left[log\left({\hat{\vartheta }}_{i}\right)\right]\right\}}{2z_{\left(1-\frac{\alpha }{2}\right)}}$$

(8) $$=\frac{log\left\{e^{log\left({\hat{\vartheta }}_{i}\right)+z_{\left(1-\frac{\alpha }{2}\right)}SE\left[log\left({\hat{\vartheta }}_{i}\right)\right]}\right\}-log\left\{e^{log\left({\hat{\vartheta }}_{i}\right)-z_{\left(1-\frac{\alpha }{2}\right)}SE\left[log\left({\hat{\vartheta }}_{i}\right)\right]}\right\}}{2z_{\left(1-\frac{\alpha }{2}\right)}}$$

(9) $$=\frac{log\left[UCI\left(\vartheta_{i}\right)\right]-log\left[LCI\left(\vartheta_{i}\right)\right]}{2z_{\left(1-\frac{\alpha }{2}\right)}},$$

where $LCI\left({\hat{\vartheta }}_{i}\right)$ and $UCI\left({\hat{\vartheta }}_{i}\right)$ are the lower and upper CIs (LCI, UCI) for$\;\left({\hat{\vartheta }}_{i}\right).$ The observant reader will notice that the last equation is equivalent to each SE term in a relative effects meta-analysis indexed by $\left(i\right)$ [4].

-Method 2

Using the formula for the lower bound of $\left({\hat{\vartheta }}_{i}\right)$ , i.e.,

(10) $$LCI\left({\hat{\vartheta }}_{i}\right)=e^{log\left({\hat{\vartheta }}_{i}\right)-z_{\left(1-\frac{\alpha }{2}\right)}SE\left[log\left({\hat{\vartheta }}_{i}\right)\right]},$$

it follows that

(11) $$log\left[LCI\left({\hat{\vartheta }}_{i}\right)\right]=log\left({\hat{\vartheta }}_{i}\right)-z_{\left(1-\frac{\alpha }{2}\right)}SE\left[log\left({\hat{\vartheta }}_{i}\right)\right]$$

(12) $$\Rightarrow SE\left[log\left({\hat{\vartheta }}_{i}\right)\right]=\frac{-\left\{log\left[LCI\left(\vartheta_{i}\right)\right]-log\left({\hat{\vartheta }}_{i}\right)\right\}}{z_{\left(1-\frac{\alpha }{2}\right)}}.$$

-Method 3

Similarly,

(13) $$UCI\left({\hat{\vartheta }}_{i}\right)=e^{log\left({\hat{\vartheta }}_{i}\right)+z_{\left(1-\frac{\alpha }{2}\right)}SE\left[log\left({\hat{\vartheta }}_{i}\right)\right]}$$

(14) $$\Rightarrow log\left[UCI\left({\hat{\vartheta }}_{i}\right)\right]=log\left({\hat{\vartheta }}_{i}\right)+z_{\left(1-\frac{\alpha }{2}\right)}SE\left[log\left({\hat{\vartheta }}_{i}\right)\right]$$

(15) $$\Rightarrow SE\left[log\left({\hat{\vartheta }}_{i}\right)\right]=\frac{log\left[LCI\left(\vartheta_{i}\right)\right]-log\left({\hat{\vartheta }}_{i}\right)}{z_{\left(1-\frac{\alpha }{2}\right)}}.$$

### 2.4. Non-Equivalence near Unity

Although the three methods for computing $\;SE\left[log\left({\hat{\vartheta }}_{i}\right)\right]$ are theoretically equivalent, imprecise rounding may yield divergent results. This is because REEs are expressed on a curvilinear logarithmic (log) scale, with the greatest departure from linearity occurring near unity. Values on a log scale are bounded by “zero and infinity” as opposed to “minus and plus infinity” on a linear scale. On a linear scale, $\left(+x\right)$ is equal in magnitude to the opposite effect of$\;\left(-x\right)$, while on the log scale, it is equal to the inverse of this value (i.e., 1/x). As REEs are centered at unity (versus zero on a linear scale), values greater than one indicate the increased occurrence of an outcome event, while those below one indicate a decreased incidence. A statistically significant result at the α-level (i.e., the probability of falsely rejecting the null hypothesis of no test effect) corresponds to a (1 − α)100% CI for an REE that does not span unity.

Because the impact of rounding is greater for values near unity, CIs for REEs should be based on the number of significant digits needed to unequivocally ascertain statistical significance rather than rounding to a fixed number of decimal places. For instance, rounding the UCI for an REE of 0.9961 to two fixed decimal places (i.e., 1.00) will confound the statistical significance of the result versus rounding to two significant digits (i.e., 0.996) (see underbars). The further an REE deviates from unity, the less impact it has on the width of the CI, as computed by the three respective methods [5].

### 2.5. Estimating Rounding Inaccuracy

For a given (i), a simple check of rounding inaccuracy may be performed by comparing $\left({\hat{\vartheta }}_{i}\right)$ with the square root of $LCI\left({\hat{\vartheta }}_{i}\right)$ times $UCI\left({\hat{\vartheta }}_{i}\right)$. This is easily seen by rearranging the formula for LCI and UCI and equating results. That is,

(16) $$LCI\left({\hat{\vartheta }}_{i}\right)=e^{log\left({\hat{\vartheta }}_{i}\right)-z_{\left(1-\frac{\alpha }{2}\right)}SE\left[log\left({\hat{\vartheta }}_{i}\right)\right]}=\frac{e^{log\left({\hat{\vartheta }}_{i}\right)}}{e^{z_{\left(1-\frac{\alpha }{2}\right)}SE\left[log\left({\hat{\vartheta }}_{i}\right)\right]}}$$

(17) $$\Rightarrow e^{z_{\left(1-\frac{\alpha }{2}\right)}SE\left[log\left({\hat{\vartheta }}_{i}\right)\right]}=\frac{{\hat{\vartheta }}_{i}}{LCI\left(\vartheta_{i}\right)},$$

and by symmetry

(18) $$UCI\left({\hat{\vartheta }}_{i}\right)=e^{log\left({\hat{\vartheta }}_{i}\right)+z_{\left(1-\frac{\alpha }{2}\right)}SE\left[log\left({\hat{\vartheta }}_{i}\right)\right]}=e^{log\left({\hat{\vartheta }}_{i}\right)}e^{z_{\left(1-\frac{\alpha }{2}\right)}SE\left[log\left({\hat{\vartheta }}_{i}\right)\right]}$$

(19) $$\Rightarrow e^{z_{\left(1-\frac{\alpha }{2}\right)}SE\left[log\left({\hat{\vartheta }}_{i}\right)\right]}=\frac{UCI\left({\hat{\vartheta }}_{i}\right)}{{\hat{\vartheta }}_{i}}.$$

(20) $$∴\frac{{\hat{\vartheta }}_{i}}{LCI\left({\hat{\vartheta }}_{i}\right)}=\frac{UCI\left({\hat{\vartheta }}_{i}\right)}{{\hat{\vartheta }}_{i}}$$

(21) $$\Rightarrow {\hat{\vartheta }}_{i}=\sqrt{LCI\left({\hat{\vartheta }}_{i}\right)\cdot UCI\left({\hat{\vartheta }}_{i}\right)}.\;\;$$

As a rule of thumb, a value of $\left({\hat{\xi }}_{n}\right)$ greater than $\left(n\right)\;times$ (0.001) would suggest an undesirable level of inaccuracy and indicate that a greater number of significant digits is needed when analyzing or presenting the data. Accordingly, a measure of the rounding inaccuracy involving $\left(n\right)$ relative effect terms may be estimated as

(22) $${\hat{\xi }}_{n}=\sum_{i}^{n}\left|{\hat{\vartheta }}_{i}-\sqrt{LCI\left({\hat{\vartheta }}_{i}\right)\cdot UCI\left({\hat{\vartheta }}_{i}\right)}\right|.$$

When $\left(n=2\right)$, the formula gives the cumulative rounding inaccuracy for risk reduction on a log difference scale when comparing two REEs. The formula also may be used in the case of multiple REE terms (i.e., n>2) to gauge the cumulative rounding inaccuracy for a meta-analysis.

### 2.6. Review of Rounding Rules

Rounding rules posit that true uncertainty “is as large as, or larger than, that predicted by the rule but of the same order of magnitude” [6]. Per se, “uncertainty is the absolute limit of error” [7]. Round-off error, vis-à-vis limiting the number of significant digits, conveys information regarding the degree of uncertainty in a measurement [8]. Ideally, rounding should be implemented in such a fashion that the process does confound the intrinsic meaning of a study. While basic rules for rounding have long existed in the scientific literature, achieving a consistent and universally agreed upon framework to minimize the propagation of round-off error has been challenging.

The application of rounding rules has traditionally centered on addition/subtraction, multiplication/division, and logarithmic/exponential operations. Extended to the field of epidemiology, this more broadly has included strategies for handling REEs and CIs, such that rounded values near unity are not subject to misinterpretation. Also, guidelines for *p*-values have been set forth to present results in the most parsimonious manner (i.e., minimizing unnecessary decimal representation while preserving accuracy).

#### 2.6.1. Addition/Subtraction and Multiplication/Division Operations

In the case of addition/subtraction, it is generally prescribed that rounded values should not be more precise than the least precise measurement, whereas for multiplication/division, results ideally should be expressed with the same number of significant figures as constituent measurements with the least number of significant digits before rounding [1]. Violations occur, for example, when a rounded mean has greater precision than any component number used to compute this value. Similarly, the rounded CI for a mean value should not have greater precision than the mean and standard deviation needed to compute the lower and upper bounds of this expression.

#### 2.6.2. Logarithmic/Exponential Operations

The error propagation equation for logarithms, by an analogy for exponential operations, may be used to estimate the number of significant digits needed for rounding. That is, for $y={log}_{b}x$, the uncertainty for $\left(y\right)$ is approximately equal to $precision\left(x\right)/log\left(b\right)$ [6].

#### 2.6.3. *p*-Values

When rounding *p*-values, zeros immediately following the decimal are considered to be “placeholders” rather than significant digits [8]. By convention, non-significant *p*-values are rounded to two or three figures beyond the decimal place. The use of *p* = NS to indicate a non-significant result or *p* < 0.05 to represent a non-specific level of significance may conceal important information and should be discouraged [9].

#### 2.6.4. Relative Effect Estimates

The lower and upper confidence limits for REEs preferably should be rounded with respect to the number of digits needed to determine a significant result [10]. This avoids the ill-advised practice of indiscriminately rounding to a fixed number of decimal places. While other rounding rules take a slightly different approach, it is generally recognized that REEs are expressed on a logarithmic (non-linear) scale and require special consideration. One alternative rule recommends that risk ratio (RR) values close to unity should be rounded to two decimal places if the underlying rate is at least 1/1000 person years and to more digits if the underlying rate is high (i.e., 1/10 py) but not exceeding the “minimal clinically important difference” [5].

On the other hand, the “Rule of Four”, which is based on the “maximum absolute fractional rounding error”, poses an upper bound on the error rate (i.e., <1.3%). Briefly, this rule entails dividing the risk ratio by four and rounding down to two significant figures, such that the final rounded result is reported to that number of decimal places [11]. A downside of the “Rule of Four” is that a UCI of 0.9973 and an LCI of 1.0042 are both rounded to 1.0, thus obscuring a statistically significant result. However, researchers are cautioned to avoid presenting “spuriously significant results” that lack clinical relevance or practical value, based only on whether or not the CI spans unity.

## 3. Examples

### 3.1. Risk Reduction

Circulating tumor cell assays (i.e., liquid biopsies) have become an important tool for the early detection and targeted treatment of cancer among at-risk populations, helping to improve patient survival. These non-invasive tests search for tumor cells that have detached from a primary cancer and have entered the circulating bloodstream. A large patient support foundation has been using a liquid biopsy assay (Kit #1) produced by Company (A) to screen for cancer. However, the foundation has learned of a potentially more sensitive assay (Kit #2) that is being offered by Company (B). The executive team at the foundation would like to move forward with switching to Kit #2, but only if there is a 50% hazard ratio (mortality) risk reduction at the α = 0.05 level of statistical significance. A randomized clinical trial was conducted, yielding the following reported results:

(23) $${\hat{HR}}_{1}=1.6,\;95\%CI=\left(1.3-2.1\right),$$

(24) $${\hat{HR}}_{2}=1.1,\;95\%CI=\left(0.86-1.3\right).$$

Taking the ratio of these HRs and rounding the computed results to three significant digits (again noting underbars, discounting zero values immediately following the decimal place) gives

(25) $$\;\;\;\frac{{\hat{HR}}_{1}}{{\hat{HR}}_{2}}=\underline{1}.\underline{45},$$

with

(26) $${CI}_{95}\left(\frac{{\hat{HR}}_{1}}{{\hat{HR}}_{2}}\right)=\left(\underline{1}.0\underline{60}-\underline{2.}\underline{00}\right)\;\left(by\;default\;SE\;approach\right);\hat{P}=0.0\underline{203}.$$

Here, the CI does not include unity, indicating a statistically significant result. However, because the risk reduction is less than 50% [i.e., (1.45−1.00 = 45%], the foundation decides against adopting Kit #2.

An independent consultant questions these results given that the analysis was conducted on values rounded to only two significant digits (yielding a cumulative inaccuracy of $\hat{\xi }\;$ = 0.055). Re-analyzing the data with greater accuracy, their corrected results give

(27) $${\;\hat{HR}}_{1}=1.6443412302,\;95\%CI=\left(1.3161806950-2.0543213327\right),$$

(28) $${\hat{HR}}_{2}=1.0774119191,\;95\%CI=\left(0.8616745800-1.3471633844\right),$$

(29) $$\frac{{\hat{HR}}_{1}}{{\hat{HR}}_{2}}=1.5261955071,$$

with

(30) $${CI}_{95}\left(\frac{{HR}_{1}}{{HR}_{2}}\right)=\left(1.1133527722-2.0921246023\right);\hat{P}=0.0086086358.$$

It is now seen that the risk reduction in Kit #2 with respect to Kit #1 is ~53% [i.e., (1.5261955071−1)], with the lower CI being noticeably further from unity (indicating a greater level of statistical significance). Based upon the more precise re-analyzed data, the foundation decided to move forward with implementing Kit #2.

### 3.2. Comparison of the Three Methods for Computing SE

Again, consider an example wherein RRs and 95% CIs were obtained from imprecisely reported secondary sources. That is

(31) $${\;\hat{RR}}_{1}=1.9,\;95\%CI=\left(1.5-2.5\right),$$

and

(32) $${\hat{RR}}_{2}=1.3,\;95\%CI=\left(1.0-1.7\right).$$

Computing the ratio of ${\;\hat{RR}}_{1}$ and ${\hat{RR}}_{2}$ gives

(33) $$\frac{{\hat{RR}}_{1}}{{\hat{RR}}_{2}}=\underline{1}.\underline{46}.$$

The respective CIs and *p*-value estimates using the three alternative SE methods are shown below.

-SE Method 1

(34) $${CI}_{95}\left(\frac{{\;\hat{RR}}_{1}}{{\hat{RR}}_{2}}\right)=\left(\underline{1}.0\underline{11}-\underline{2}.\underline{11}\right);\;\hat{P}=0.0\underline{434}.$$

-SE Method 2

(35) $${CI}_{95}\left(\frac{{\;\hat{RR}}_{1}}{{\hat{RR}}_{2}}\right)=\left(\underline{1}.0\underline{27}-\underline{2}.\underline{08}\right);\hat{P}=0.0\underline{352}.$$

-SE Method 3

(36) $${CI}_{95}\left(\frac{{\;\hat{RR}}_{1}}{{\hat{RR}}_{2}}\right)=\left(0.99\underline{957}-\underline{2}.\underline{15}\right);\;\hat{P}=0.0\underline{526}.$$

Noticeably, each method for estimating the SE yields contradictory CIs and *p*-value estimates. The null hypothesis for this example is rejected using SE Methods 1 and 2 but not for SE Method 3 (which fails to achieve a statistically significant result, given  α=0.05).

### 3.3. Meta-Analysis

In a meta-analysis, RRs and 95% CIs were obtained from eight studies, each assessing the association between an environmental exposure and a subsequent irregular heart condition. The reported pooled estimate and *p*-value was suggestive of a significant effect among patients exposed (versus not exposed) to the risk factor. However, the meta-analysis was performed on study-specific risk estimates that were obtained from the literature and provided to only two decimal places, raising questions about the rounding inaccuracy of the findings. Referring to Table 1, only source studies #2 and #7 had an acceptable rounding inaccuracy that was less than the “rule of thumb” cutoff value of 0.001. Additionally, the cumulative rounding inaccuracy was ~3-fold higher than the composite cutoff value of 0.008. This submits that the point estimates for one or more of the individual studies should have been reported with more precision to ensure greater confidence in the meta-analytic results. When possible, it may be necessary to contact the authors of the original source studies to obtain this information.

### 3.4. Data Truncation

Similar to rounding error, data truncation involves disregarding small fractional values that may accumulate when performing complex analytical operations. The propagation of truncation error can result in less precise and accurate conclusions. The conceptual example below, involving HRs, illustrates how seemingly trivial data truncation in theory can yield illogical and counterintuitive findings [10].

Let $\left({\hat{HR}}_{1}\right)$ and $\left({\hat{HR}}_{2}\right)\;$ denote two “bounded” estimates that are close to but not exactly equal to one another. That is, when the event rate for the referent group (*P*_0_) of an HR under consideration in this example is greater than 0.05 and Cohen’s d is less than or equal to 0.80 (large effect size), the HR will not exceed a bounded value of 10 [12]. Furthermore, consider $\left(\gamma \right)$ to be a positive, real-valued number that is near zero and represents truncation error. Abstractly, this may be written as

(37) $${\hat{HR}}_{1}=\left({\hat{HR}}_{2}+\gamma \right),\;∋:\;\left[\left\{\gamma <<1\;\right\}\wedge \;\left\{{\hat{HR}}_{1,2}\leq 10\right\}\wedge \;\left\{{\hat{HR}}_{1,2},\;\gamma ,\;\left|{\hat{HR}}_{1}-{\hat{HR}}_{2}\right|>0\right\}\right].$$

Multiplying each side by$\;\left({\hat{HR}}_{1}\right)\;$gives

(38) $${\hat{HR}}_{1}^{2}={\hat{HR}}_{1}\left({\hat{HR}}_{2}+\gamma \right)$$

(39) $$\Rightarrow {\hat{HR}}_{1}^{2}={\hat{HR}}_{1}{\hat{HR}}_{2}+{\hat{HR}}_{1}\gamma$$

(40) $$\Rightarrow \left({\hat{HR}}_{1}^{2}-{\hat{HR}}_{2}^{2}\right)=\left({\hat{HR}}_{1}{\hat{HR}}_{2}-{\hat{HR}}_{2}^{2}\right)+{\hat{HR}}_{1}\gamma .$$

Since by definition $\left(\gamma \right)$ is negligible and $\left({\hat{HR}}_{1}\right)$ has a small finite upper bound of less than or equal to 10, one may reasonably truncate the right-hand side of the above equation to $\left({\hat{HR}}_{1}{\hat{HR}}_{2}-{\hat{HR}}_{2}^{2}\right).$ Thus,

(41) $$\left({\hat{HR}}_{1}^{2}-{\hat{HR}}_{2}^{2}\right)\approx \left({\hat{HR}}_{1}{\hat{HR}}_{2}-{\hat{HR}}_{2}^{2}\right)$$

(42) $$\Rightarrow \left({\hat{HR}}_{1}+{\hat{HR}}_{2}\right)\left({\hat{HR}}_{1}-{\hat{HR}}_{2}\right)\approx {\hat{HR}}_{2}\left({\hat{HR}}_{1}-{\hat{HR}}_{2}\right)$$

(43) $$\Rightarrow \left({\hat{HR}}_{1}+{\hat{HR}}_{2}\right)\approx {\hat{HR}}_{2}$$

(44) $$\Rightarrow \left({\hat{HR}}_{2}+\gamma +{\hat{HR}}_{2}\right)\approx {\hat{HR}}_{2}$$

(45) $$\Rightarrow \left(2{\hat{HR}}_{2}+\gamma \right)\approx {\hat{HR}}_{2}.$$

Again, because $\left(\gamma \right)$ is very small, the left-hand side of the above equation may be truncated to $\left(2{\hat{HR}}_{2}\right).$ That is,

(46) $$\left(2{\hat{HR}}_{2}\right)\approx {\hat{HR}}_{2}.$$

(47) ∴2≈1.   Q.E.D.

By twice allowing truncation of the data, the propagated error yields the paradoxical result that “2 is approximately equal to 1”. Thus, truncation error cannot simply be disregarded, especially in applications where the loss of accuracy is additive or multiplicative. In such cases, overlooking the accumulation of truncation error can significantly affect the computational accuracy of results.

### 3.5. Floating-Point Chopping Error

Modern computer operating systems generally represent real numbers in a floating-point format, consisting of a sign, exponent, and mantissa [13]. A type of truncation or rounding error, known as floating-point chopping, typically occurs when the floating-point representation of a number exceeds the memory capability of a computer. Floating-point chopping can particularly bias epidemiologic analyses when the REEs under study greatly differ in magnitude.

Consider two independent REEs $\left({\hat{\vartheta }}_{1}\;and\;{\hat{\vartheta }}_{2}\right)$ from a covariate-adjusted observational study. The aim is to assess the association between adverse pregnancy outcomes and exposure to ionizing radiation (versus non-exposure), with respect to a common referent control group. In this scenario, the first group are residents living adjacent to a radioactive waste dump, and the second are members of an organic farming community, while the common referent control group consists of individuals residing in an average mid-size town. To be expected, the ratio of REEs in such an example will be quite large with the potential of computational floating-point error to occur. As illustrated below, floating-point error can increase dramatically as ${\hat{\vartheta }}_{1}\;and{\hat{\vartheta }}_{2}$ deviate from one another.

Consider the following equation, which in theory is equal to zero [14]:

(48) $$\zeta ={\left(\sqrt{{\hat{\vartheta }}_{1}+{\hat{\vartheta }}_{2}}-\sqrt{{\hat{\vartheta }}_{1}}\right)}^{-1}-\frac{\sqrt{{\hat{\vartheta }}_{1}+{\hat{\vartheta }}_{2}}+\sqrt{{\hat{\vartheta }}_{1}}}{{\hat{\vartheta }}_{2}}.$$

**Figure 1:**
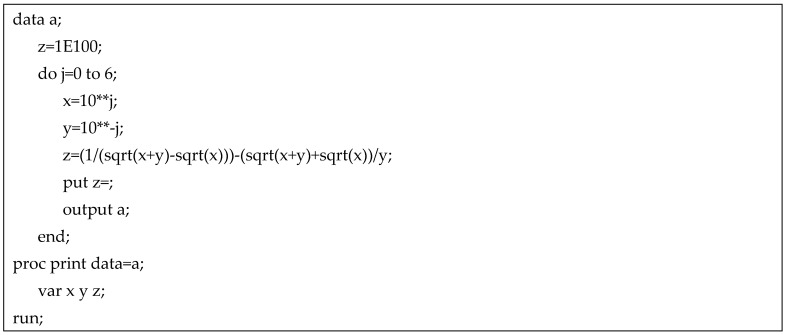
SAS code for floating-point chopping example shown in Table 2.

Here, $\left(\zeta \right)$ reflects the degree of floating-point chopping error, with bias depicted as the distance from zero. Referring to Table 2 (see Figure 1 for SAS code), it is seen that $\left(\zeta \right)$ increases as ${\hat{\vartheta }}_{1}\;and\;{\hat{\vartheta }}_{2}$ diverge in magnitude. Furthermore, note the negative value of $\left(\zeta \right)$ in row 1 and the peculiar jump in the magnitude of $\left(\zeta \right)$ beyond row 5. This type of discrepancy is largely attributable to a suboptimal floating-point library and can be mitigated by increasing the number of useable registers. Floating-point chopping error also can be prevented by restricting the study design to offset disparate REE values. In practice, $\left(\zeta \right)$ can similarly be computed for real-life values of $\left({\hat{\vartheta }}_{1}\;and\;{\hat{\vartheta }}_{2}\right)$ to check for possible floating-point chopping error, although the interpretation of the result is to a certain degree subjective and contingent on the specifics of the problem at hand. That is, a large value for $\left(\zeta \right)$ that is critical in one application may be irrelevant in a different situation and vice versa.

### 3.6. Exponentially Diminished Relative Effect Estimates

An REE with a value slightly above unity would imply a trivial or even ignorable increase in the occurrence of an outcome event. However, raising this effect to a large power can have exponential consequences. For instance, an OR of 1.01, inflated to a power of 1000, would be approximately equal to 20,959. By disregarding the fractional part of the base, the result is diminished to a null value (i.e., 1.00^1000^ = 1.00). That is, a small truncation effect of only one percent in the OR greatly impacted the outcome interpretation of this equation.

## 4. Discussion

### 4.1. Overview

Rounding has important implications for how the findings of an analysis are interpreted. The objective of rounding is to preserve “closeness to truth” and the “repeatability” of results while achieving an acceptable degree of parsimony. This concept underlies the “Goldilocks” principle of rounding, i.e., “not too much and not too little” [11]. When one deviates from the Goldilocks’ principle, rounding can result in less precise and accurate conclusions, and the propagation of rounding error can yield illogical and counterintuitive findings. Although accuracy and precision represent two important aspects of rounding error, they are not necessarily mutually exclusive nor independent. Hence, erroneous results may be either inaccurate and imprecise, precise but inaccurate, or accurate but imprecise.

The focus of the current manuscript was to quantify the effect of rounding error (also referred to as round-off error) on epidemiologic studies involving REEs. Round-off error was defined as inexactness or uncertainty occurring in the computation and presentation of results [1]. This is particularly relevant given the increasingly complicated methods that underlie epidemiological analyses. The propagation of seemingly trivial round-off error involving multiple sequential operations can have difficult-to-predict consequences—potentially distorting the interpretation of study results. A useful tool for estimating the inaccuracy of an REE rounded to too few significant digits was derived. This approach is handy to gauge bias when estimating the logarithmic risk difference between two groups or in more complex cases involving pooled REEs (e.g., meta-analysis). Additionally, a simple equation was provided to compute the relative magnitude of floating-point chopping error.

### 4.2. Rounding Error Propagation

As a means to streamline computations, researchers may inadvertently allow for the use of low-precision rounded values in an epidemiological analysis, leading to the propagation of rounding error. This often is the case when using REEs based on secondhand rounded values obtained from the literature.

The meta-analysis of several epidemiologic reports involving rounded effect estimates poses an important illustration of such error propagation. Similar to risk reduction on a log difference scale, wherein SE terms may be determined from rounded REEs and associated CIs obtained from published studies, a meta-analysis is likewise prone to this type of bias. A commonly used guideline in practice is that these point estimates are given to “two significant figures” [4]. However, for a meta-analysis that is conducted on a large number of constituent studies, the accumulated rounding error can seriously bias the end result, raising concerns about using data rounded to only two significant digits. While acceptable as an instructional “textbook” exercise, such imprecise rounding frequently leads to error propagation and is best circumvented in real-world applications. Ideally, the cumulative rounding error inaccuracy $\left(\hat{\xi }\right)$ should be computed when performing an epidemiological analysis entailing multiple REEs, such as a meta-analysis. This will help to gauge if a sufficient number of significant digits has been used when rounding values and also serves as a guide for the presentation of rounded values.

### 4.3. Limitations

A risk reduction ratio reflects a combined error estimate of six parameters. Thus, pinpointing a specific source of rounding inaccuracy may not be explicit in the computation of$\;\left(\hat{\xi }\right)$. In some situations, $\left(\hat{\xi }\right)$ may be unduly influenced by a single parameter yet manifest as an unacceptable overall indication of rounding error. A feasible solution is to partition $\left(\hat{\xi }\right)$ to focus on the rounding error of each component, respectively. Another potential concern is the implicit assumption that sample sizes for each group are reasonably large, with comparable round-off error rates. Barring premature patient withdrawals and dropouts, this usually is not an issue for a balanced randomized clinical trial but may be a concern when analyzing retrospective data with unbalanced groups. Also, a “one-fit-all” rule for rounding implies that each group in an analysis will be treated equally, regardless of sample size or the need to round with more or less precision, respectively [15].

Another important limitation is that unrounded electronic data may not always be available, and analysts may need to rely on imprecise parameter estimates obtained from published sources. Or it may be deemed too impractical, from an illustration standpoint, to work with unrounded data [3,16,17]. Again, risk reduction estimation and meta-analyses are classic examples in this respect. In certain cases, it may be acceptable to forego a high degree of significant digits, providing that they are “effectively unrounded data” in the interpretation of analytic results [18]. Otherwise, one should avoid the use of rounded values in intermediate epidemiological computations and only round results at the end stage for presentation purposes [19]. Yet, the propagation of round-off error (or data truncation) is frequently implicit to high-dimensional analyses involving complex matrix operations and multi-step algorithms. Fortunately, the availability of arbitrary precision arithmetic has greatly reduced this concern, although some analytic software packages still lack this capability [20].

### 4.4. Practical Advice

Rounding provides a convenient tool for the succinct presentation of complex epidemiological analyses. The goal is to report the minimal number of significant digits needed to convey accuracy without under- or over-stating the inherent precision of the data (i.e., minimum adequate accuracy versus maximum available accuracy) [21]. In practice, using fewer significant digits to express values may introduce systemic bias and impede the ability to reliably estimate variability [18,19]. This is especially true in multifaceted analyses, where round-off error may be magnified and yield “diluted or untrue findings” [1]. Generally speaking, “the more certain or accurate a number, the more significant figures that must be used to convey that level of accuracy” [22].

The conceptual examples presented in this manuscript are intended to illustrate rounding errors that theoretically may arise in epidemiological studies. Although such errors in real-world practice tend to be nominal and only occasionally sway conclusions to a significant degree, it remains prudent in research practice to carefully monitor their occurrence. Particularly in complex situations with the potential for rounding error propagation, power and sample size computations may need to be adjusted accordingly in the design stage of a study or accounted for when analyzing the data. While information is limited in this respect, the interested reader is referred to appropriate sections on misclassification bias and measurement error in classical epidemiology textbooks for guidance in this matter.

## 5. Conclusions

The objective of rounding is to convey a truthful yet parsimonious outcome. However, rounding inaccuracies, especially when accumulated, can lead to the misrepresentation of research findings, whether intentional or not. Thus, one must be attentive to the inappropriate rounding of numbers to avoid erroneous results and misguided conclusions.

The ability to quantify rounding inaccuracies, as highlighted in the current manuscript, provides an important tool for interpreting the validity and trust of an epidemiological analysis.

## Figures and Tables

**Table 1:** Meta-analysis rounding inaccuracy.

Source Study (i)	$\hat{RR}$ (95%CI)	Rounding Inaccuracy $\left({\hat{\xi }}_{i}\right)$
1	1.29 (1.01–1.66)	0.0048358969
2	2.32 (1.07–5.03)	0.0000646561
3	1.03 (1.01–1.04)	0.0051097620
4	1.67 (1.20–2.32)	0.0014677108
5	1.30 (1.00–1.69)	0.0077152017
6	1.67 (1.40–2.00)	0.0033200531
7	1.86 (1.39–2.49)	0.0004031821
8	1.12 (1.07–1.17)	0.0011166281
Cumulative Rounding Inaccuracy $\left(\sum_{i}{\hat{\xi }}_{i}\right)$		0.0240330908

CI = confidence interval. $\hat{RR}$ = risk ratio estimate.

**Table 2:** Float-point chopping error †.

${\hat{\vartheta }}_{1}$	${\hat{\vartheta }}_{2}$	Floating-Point Chopping Error $\left(\zeta \right)$
1	1	−0.000000000000000444089
10	0.1	0.000000000001172396
100	0.01	0.0000000004260983
1000	0.001	0.000001643195
10,000	0.0001	0.0000495147
100,000	0.00001	201.13583846
1,000,000	0.000001	21,151.024511

^†^ Computed using SAS v9.4 (TS1M7) on a X64 DSRV19 platform.

## Data Availability

Data are contained within the article.
